# Identifying Degenerative Brain Disease Using Rough Set Classifier Based on Wavelet Packet Method

**DOI:** 10.3390/jcm7060124

**Published:** 2018-05-28

**Authors:** Ching-Hsue Cheng, Wei-Xiang Liu

**Affiliations:** Department of Information Management, National Yunlin University of Science and Technology, Yunlin 64002, Taiwan; d10223002@yuntech.edu.tw

**Keywords:** degenerative brain disease, rough sets, segmentation, magnetic resonance imaging, wavelet packet

## Abstract

Population aging has become a worldwide phenomenon, which causes many serious problems. The medical issues related to degenerative brain disease have gradually become a concern. Magnetic Resonance Imaging is one of the most advanced methods for medical imaging and is especially suitable for brain scans. From the literature, although the automatic segmentation method is less laborious and time-consuming, it is restricted in several specific types of images. In addition, hybrid techniques segmentation improves the shortcomings of the single segmentation method. Therefore, this study proposed a hybrid segmentation combined with rough set classifier and wavelet packet method to identify degenerative brain disease. The proposed method is a three-stage image process method to enhance accuracy of brain disease classification. In the first stage, this study used the proposed hybrid segmentation algorithms to segment the brain ROI (region of interest). In the second stage, wavelet packet was used to conduct the image decomposition and calculate the feature values. In the final stage, the rough set classifier was utilized to identify the degenerative brain disease. In verification and comparison, two experiments were employed to verify the effectiveness of the proposed method and compare with the TV-seg (total variation segmentation) algorithm, Discrete Cosine Transform, and the listing classifiers. Overall, the results indicated that the proposed method outperforms the listing methods.

## 1. Introduction

With a strengthened sanitary environment and effective antibiotics, death rates have decreased since the twentieth century. The average life expectancy of the global population has generally increased; therefore, the aging population has become a serious problem. The major disease pattern has also changed to “age-related degenerative diseases”. Medical issues related to degenerative brain diseases, such as Alzheimer’s disease (AD) and mild cognitive impairment (MCI), have gradually raised concerns.

Magnetic resonance imaging (MRI) is a noninvasive diagnostic technique that produces computerized images of internal body tissues and is based on nuclear magnetic resonance of atoms within the body induced by the application of radio waves. MRI is an essential technique used to identify patients with Alzheimer’s disease. By scanning the brains of people not diagnosed with Alzheimer’s disease, greater levels of atrophy in a specific part of the cerebral cortex may be observed, which is associated with a threefold increase in developing Alzheimer’s disease [1]. Nuclear Magnetic Resonance Imaging (NMRI) is also known as spinning imaging and Magnetic Resonance Imaging (MRI). The majority of body tissues can be examined using MRI, which is especially clear and delicate when imaging soft tissue; therefore, this technology surpasses any other current medical imaging systems. Much research has used MRI to examine issues related to brain degenerative diseases. For example, MRI of Alzheimer’s patients was also used in texture analysis [2]. In addition, MR images of mild cognitive impairment were applied to conduct pattern classification [3].

Human tissues are composed of 75% water molecules, and hydrogen atoms have flexibility and signal strongly in nuclear magnetic resonance; therefore, MRI is regarded as a first choice for imaging elements. In addition, MRIs have the following three additional advantages: (1) excellent imaging resolution of soft tissues, (2) non-invasive and (3) non-radioactive; therefore, it has become a popular brain imaging technique. Based on these advantages, MRI was used in this study.

Image segmentation is an important pre-processing step. The purpose of image segmentation is to separate the desired elements from the elements with similar properties in the image [4]. Generally, medical image segmentation is performed by specialists, and different specialists will produce different segmentation results based on their past experiences and training process. In addition, the manual segmentation process is time-consuming and laborious. Furthermore, the proposed results of the same image may be different due to the specialist’s judgment of the mental condition.

In image segmentation methods, numerous automatic and semi-automatic segmentation methods were developed by researchers. In general, the automatic segmentation method does not require any human interaction, which saves time. However, several restrictions regarding automatic segmentation exist. Full automatic segmentation may be categorized into two main types for use in different applications: (1) applied to a specific type of image and (2) applied to general types of images [2]. In contrast to the automatic segmentation methods, semi-automatic segmentation applies the same segmentation method to any image without restrictions on the type [5].

The gray-level values of abnormal brain tissues are quite different from normal tissues in volume and intensity. In addition, the characteristics of different brain images will increase the complexity of the segmentation; therefore, the identification of an interesting area in the images is a challenging task and is also a critical step following feature selection and classification.

The most frequently used medical image segmentation techniques are classified into the following five categories: (1) the region-based method, (2) the thresholding-based method, (3) the edge-based method, (4) the classification-based method, and (5) hybrid techniques. However, each method has its disadvantages and restrictions on the segmentation process; therefore, the concept of hybrid techniques was proposed by several researchers. For example, the hybrid techniques that integrate the results of boundary detection and region growth are expected to provide more accurate segmentation of the images [6].

Based on these existing problems, the current segmentation method and the severity of degenerative brain diseases, as well as assisting medical staff in judging and classifying degenerative brain diseases, are important issues that need to be further explored. Therefore, this study proposed to develop a hybrid semi-automatic segmentation algorithm as the pre-processing step to improve the restriction in the automatic segmentation process and to enhance the segmentation performance. The results will combine the wavelet packets and rough sets to enhance classification accuracy.

The paper is organized as follows: Section 2 provides materials and methods including the related works and the proposed method. Section 3 presents experiments and results. Section 4 presents the findings. Lastly, Section 5 provides conclusions and suggests future studies.

## 2. Materials and Methods

This section will briefly review the related literature including segmentation techniques, wavelet packet transform, and rough sets. The proposed method and the proposed algorithm are introduced in the following.

### 2.1. Segmentation Techniques

The common MRI segmentation techniques are divided into five categories: the region-based method; the thresholding-based method; the edge-based method; the classification-based method; and the hybrid techniques. A region-based method such as the region-growing algorithm begins with a seed point through the examination of the average gray-level, the texture, and other properties. Ultimately, using this method, the pixels with similar properties will be included in each area. The region-based method, such as the region growing algorithm, was used by other researchers [7].

The thresholding-based method distinguishes the object and the background primarily based on setting a threshold value. The common thresholding-based segmentation methods are dual thresholding and adaptive thresholding. The dual thresholding method primarily relies on two threshold values and distinguishes the objective from the background based on the two values. The two threshold values are unable to perform well with complex images; therefore, adaptive thresholding divides the image into several sub-regions and sets the threshold values to each region. However, these two methods are inefficient for images that blur at object boundaries or for multiple image component segmentation [8].

Edge-based algorithms perform the segmentation process with multiple types of edge detection filters, such as the Sobel and the Laplace detectors. However, the derivative nature of this approach causes these detectors to be extremely sensitive to image noise levels [9].

The classification-based algorithms consist of the self-organizing map (SOM) and the fuzzy c-means (FCM). The classification-based method needs to train first, and its performance primarily depends on the training parameters and the classifier inputs [10]. The hybrid techniques [3] integrate boundary detection and the region-growing method to obtain better segmentation results. In this study, the proposed segmentation algorithm is based on hybrid techniques. The following subsection will describe the other techniques we applied in the proposed method.

- Morphological operation

Dilation and erosion are two basic morphological operations. Dilation expands the boundaries of the object for a number of pixels. In contrast to dilation, erosion is used to shrink the boundaries of the object. The morphological operation of the opening involves the above two operations. It performs the erosion first to eliminate all undesired points or lines, and subsequently uses dilation to recover the original image. If A and B are sets of the space, the opening of A by B implies that the erosion of A will follow the dilation of B. The operation performs the erosion first; therefore, the slim or the narrow parts of A will be cut, and the dilation will be performed to smooth the contour of A. Ultimately, smoothing the contour and removing small protrusions are the purposes of the opening operation.

It can be defined as Equation (1):

(1) A o B=(A⊖B)⨁B

- Sobel Filter

The Sobel filter is the common template in edge detection. Compared to other edge operators, the Sobel has two primary advantages. First, after the introduction of the average factor, this filter smooths the random noise of the image. Second, the Sobel filter is the differential of two rows or two columns; therefore, the elements of the edge on both sides are enhanced, causing the edge to appear thick and bright [11]. In addition, the Sobel operations weigh the image intensities; therefore, the effect is superior to the effects obtained using the Prewitt operation.

Two operations exist in the Sobel operator. One operation detects the edge of the vertical direction, and the other operation detects the horizontal edge. A 3 × 3 matrix is utilized and we use Q_x_ and Q_y_ to represent the vertical convolution template and the horizontal convolution template, respectively, as shown in Equation (2).

(2) $$Q_{x}=\left[\begin{array}{ccc} -1 & 0 & 1\\ -2 & 0 & 2\\ -1 & 0 & 1 \end{array}\right]\;Q_{y}=\left[\begin{array}{ccc} 1 & 2 & 1\\ 0 & 0 & 0\\ -1 & -2 & -1 \end{array}\right]$$

The Sobel operations are applied separately to the input image to produce gradient component measurements in each direction [12]. Suppose A is the original image, every point in image A should use the two operations to conduct convolution, and the results after vertical and horizontal convolution are shown in Equation (3). For every point, the calculation result after Q_x_ and Q_y_ will be added and stored in another map to confirm that the change of the edge in both directions has occurred.

(3) $$S_{x}=\left[\begin{array}{ccc} -1 & 0 & 1\\ -2 & 0 & 2\\ -1 & 0 & 1 \end{array}\right]\times A\;S_{y}=\left[\begin{array}{ccc} 1 & 2 & 1\\ 0 & 0 & 0\\ -1 & -2 & -1 \end{array}\right]\times A$$

### 2.2. TV-seg Algorithm

Total variation (TV-seg) [13] is an interactive image segmentation algorithm that is publicly available online. Users are able to use TV-seg as the segmentation tool to extract the object from an image. This algorithm uses the concept of the Geodesic Active Contour model [14] and minimizes the Geodesic Active Contour energy. The two primary steps involve identifying a binary label and dividing the image into the foreground and the background. If necessary, alpha-matting will occur along the border of the binary segmentation from the first step.

In the binary segmentation step, many brushes with different constraints will enable users to interact with the TV-seg algorithm [13]. In this step, the author minimized the following model as shown in Equation (4) to achieve the goal:

(4) $$\underset{u\in \left|0,1\right|}{\min}\left\{E_{Seg}=\int_{\Omega }g\left(x\right)\left|\nabla u\right|d\Omega +\int_{\Omega }\lambda \left(x\right)\left|u-f\right|d\Omega \right\}$$

where *u* ∈ [0,1], *d*Ω denotes image domain Ω, 0 ≤ *λ*(*x*) < ∞, *f* ∈ [0,1], when foreground *f* = (1) and background *f* = (0).

The binary segmentation result obtained in the first step is used as input for the matting step by calculating a region around contour C where matting will be performed [13].

### 2.3. Discrete Wavelet Packet Transform

The wavelet concept was proposed by Haar in 1910 [15]. Until 1986, Meyer [16] proposed the discrete wavelet transform, and Mallat [17] introduced the concept of multi-resolution to the wavelet and constructed the wavelet representation. Thus, the wavelet theory has remained important to this process.

The wavelet decomposition functions at level *m*, and time location *t_m_* can be expressed as Equation (5):

(5) $$d_{m}\left(t_{m}\right)=X\left(t\right)\Psi_{m}\left(\frac{t-t_{m}}{2^{m}}\right),$$

where *Ψm* is the decomposition filter at frequency level *m*. The effect of the decomposition filter is scaled by the factor 2*^m^* at stage *m*, but the shape is the same at all scales [18].

Wavelets have a varying window size, being wide for the slow frequencies and narrow for the fast frequencies, which is a primary advantage and leads to an optimal time–frequency resolution in all of the frequency ranges [19].

Wavelet packet analysis is an extension of the discrete wavelet transform (DWT) [20]. The wavelets decompose the low-frequency components; therefore, the wavelet packet is used to provide a wider range of possibilities for the time-frequency plane. During wavelet packet analysis, the signals are divided into four sub-bands in the first level that include one approximation and three detail coefficients. In the second level, the approximation and detail obtained from the first level will decompose further. Wavelet packet analysis combines the different levels of decomposition to achieve the optimum time–frequency representation of the original, which is an advantage of this process [19]. Therefore, based on the sufficient computer hardware resources currently available, wavelet packet analysis has been discussed and used widely.

### 2.4. Rough Sets Theory

The rough sets theory was first proposed by Pawlak [21], and it is a very useful tool for decision support systems, especially in dealing with imprecise, uncertain, and vague information during a decision process [22].

The rough sets theory uses the lower and upper approximations to define all sets and conducts the data analysis without any a priori assumptions or extra information regarding the relevant data. The rough sets theory can be described by a specific mathematical formula; therefore, vague and uncertain elements can be calculated using this theory.

In using the rough sets (RS) process, one starts with a relational database, a table of objects with attributes, and an attribute value for each object. One of the attributes is adopted as the decision-attribute, and the remaining attributes are used as conditional attributes [22].

With each subset *X* ⊆ *U*, the lower approximation and the upper approximation are presented in the following equivalent form as shown in Equations (6) and (7). The boundary region is defined as the difference between the lower approximation and the upper approximation as shown in Equation (8). The accuracy of the approximation of A by R is defined as Equation (9).

(6) $$\underline{R}X=\left\{x|{\left[x\right]}_{R}\subseteq X\right\}$$

(7) $$\bar{R}X=\left\{x|{\left[x\right]}_{R}\cap X\neq ø\right\}$$

(8) $$RN_{R\left(X\right)}=\bar{R}X\;-\;\underline{R}X$$

(9) $$\mu_{R}\left(A\right)=\frac{\sum card\;\underline{R}X_{i}}{\sum card\;\bar{R}X_{i}}$$

Rough sets have several advantages: (1) rough sets analyze inconsistent and incomplete information, (2) rough sets determine the concept and the mode that is simple and easy to operate, (3) rough sets handle inaccurate and ambiguous situations, including deterministic and nondeterministic cases, and (4) rough sets generate precise rules that are easily verified and confirmed.

### 2.5. The Proposed Method

Two major problems exist in medical image segmentation. First, the automatic segmentation method is restricted on specific image contours, but most images have different shapes and contours due to the different shooting angle. Therefore, the fully automatic segmentation method cannot be used to handle this problem. Second, the hybrid techniques integrate different methods to improve the original shortages; therefore, the performance of the single method is worse than the hybrid techniques method. Ultimately, this paper proposed a hybrid semi-automatic segmentation algorithm to solve these two problems. The proposed algorithm integrates the region-growing algorithm, the Sobel edge detection, and morphology operations to segment the brain ROI during image pre-processing. The morphology operation parameters add human recognitions to assist and enhance the performance and allow the proposed algorithm to be more suitable for different brain image contours.

Based on the above reasons, this study proposes a three-stage method based on the proposed hybrid semi-automatic segmentation algorithm to enhance the classification accuracy. The segmentation results will then use the wavelet packet to decompose and calculate the feature values. Finally, a robust classifier, rough sets, will be used to classify the images into normal and abnormal. Furthermore, TV-seg-based semi-automatic segmentation will be compared to the proposed hybrid semi-automatic segmentation algorithm. In addition, Discrete Cosine Transformation will also compare the decomposition ability of the discrete wavelet packet transform. Finally, the experimental results create a preliminary verification system on the feasibility of the proposed method.

**Figure 1:**
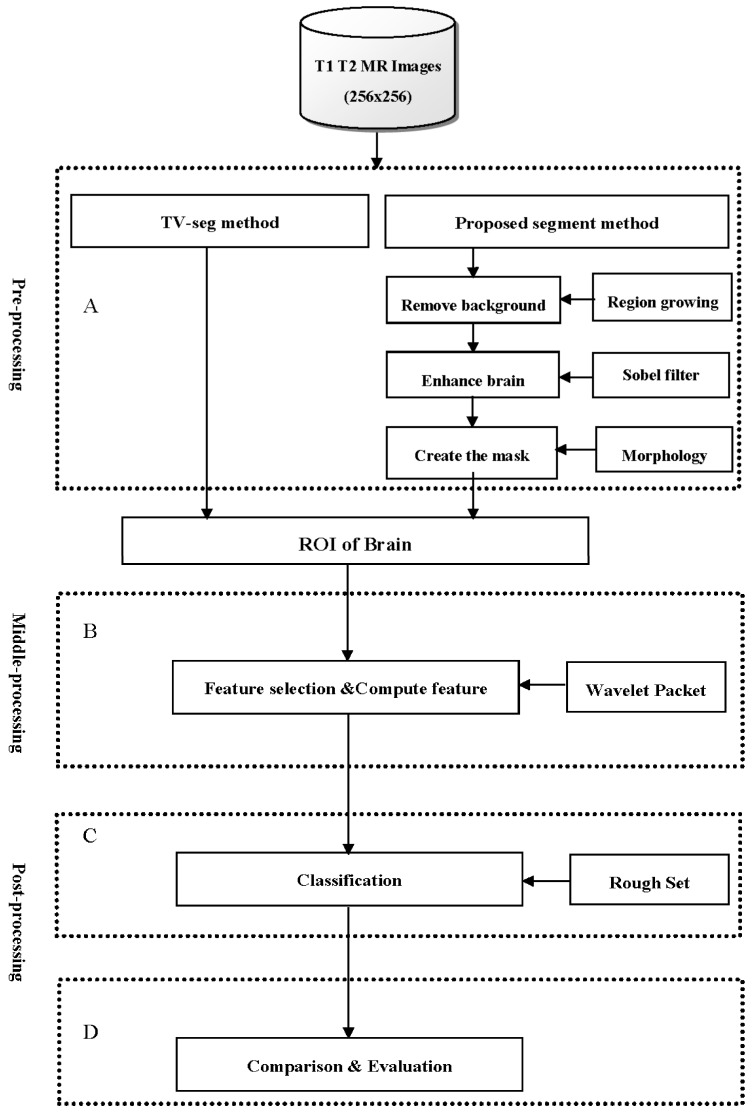
The procedure of proposed method. (**A**) a segmentation block, (**B**) an image transformation block, (**C**) a classification block, and (**D**) a comparison block.

To further understand the proposed method, this study used an architecture diagram to present the procedure of the proposed method as in Figure 1. The diagram consists of four primary blocks: (A) a segmentation block, (B) an image transformation block, (C) a classification block, and (D) a comparison block. In block A, the input brain dataset was segmented by TV-seg semi-automatic segmentation, and the proposed hybrid semi-segmentation algorithm as the image pre-processing. In block B, the brain ROI used a 2D wavelet packet to conduct the image decomposition and calculate the feature values. In block C, the rough sets LEM2 algorithm was used to classify the calculation result from block B. In block D, the following three comparison steps were applied: (1) the classification result of the proposed segmentation algorithm was compared to the TV-seg semi-automatic segmentation algorithm; (2) the Discrete Wavelet Packet Transform (DWPT) was compared to the Discrete Cosine Transform (DCT) [23,24]; and (3) the listing classifiers were then used to compare with performance of the rough sets. Finally, the comparison results were employed to evaluate and verify the availability of the proposed method.

### 2.6. The Proposed Algorithm

The proposed hybrid semi-automatic segmentation algorithm contains eight steps as follows:

**Step 1:** Input the brain MRI.

Firstly, input the T1-weight brain MR image *B*_0_ is inverted, and the image pattern is converted from RGB to GRAY for the next processing step of the region-growing algorithm.

**Step 2:** Remove the background.

In this step, the region-growing algorithm will conduct to separate the object from the background. The seed pixels were selected at (30, 30).

**Step 3:** Enhance the edge of brain contour.

In this step, two methods are used to enhance the brain contour: the Sobel filter and the adaptive thresholding method. In the experimental process, the adaptive thresholding method was found to be more suitable for the processing of the images with darker cerebrum region and obvious contours. After many experiments, the Sobel filter is more suitable for the majority of images that selected in this study.

**Step 4:** Create the mask of the cerebrum region.

In this step, the brain contour enhanced from step 2 will be used to create a binary mask image. Next, we will use the morphology operations to repair images. In this study, binary morphological opening operations and dilated operations will be used to remove the skull and fragmented images, and the output mask image is M.

**Step 5:** Obtain the brain region of interest (ROI).

**Figure 2:**
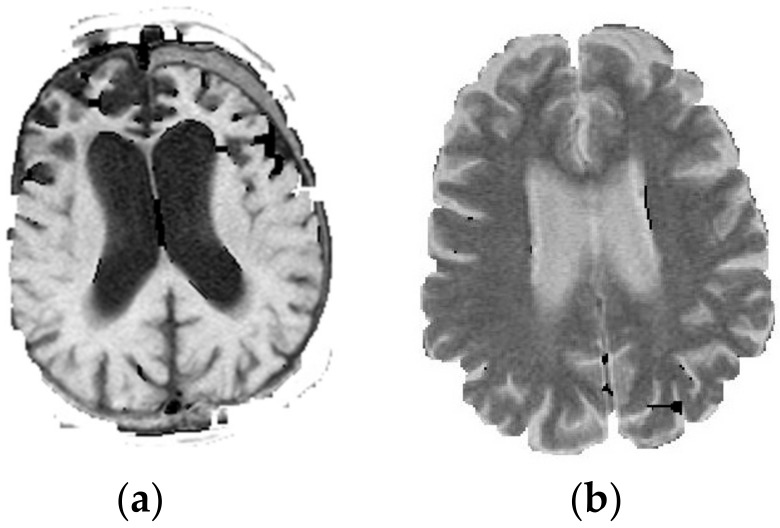
(**a**) The T1 brain region of interest (ROI); (**b**) The T2 brain region of interest (ROI).

In this step, the original image *B*_0_ will conduct the pixel to pixel multiplication with the mask *D*. The expression is shown as Equation (10), and the brain ROI is obtained from this step, and the outcome is shown in Figure 2. After the brain ROI is obtained, the following step will proceed into the middle-processing of the proposed method (in Figure 1).

(10) $$B=B_{0}\;\cdot \;D$$

**Step 6:** Wavelet packet decomposition.

In this step, the bior2.2 wavelet filters in the discrete wavelet packet transform (DWPT) are selected to decompose the brain images. The depth in the wavelet packet transform denotes the tree level, and it will also determine the number of DWPT coefficients. In this study, the depth d = 1 and d = 2 are used. If the depth d = 1, then 4 coefficients are generated. If d = 2, the image will be decomposed to 16 coefficients. Thus, in this study, the total number of DWPT coefficients is 20.

**Step 7:** Feature selection and value calculation (DWPT).

In this step, the feature values of the wavelet packet will be selected and computed. The method of feature selection is according to Avci [25], and the author used the statistical value as the input for the texture classification. In this study, the nine features include: the mean value, the median value, the maximum value, the minimum value, the range value, the mode value, the standard deviation, the median absolute value, and the mean absolute value.

Steps 6 and 7 involve the transformation step in the proposed method. In addition to conducting the DWPT decomposition, another common image transformation method, the Discrete Cosine Transform (DCT) will also apply to compare the proposed method in this stage. The selected nine features were calculated the same as the DWPT. The detailed procedure of the proposed discrete wavelet packet transform is presented in Algorithm 1.

**Algorithm 1.** Feature calculation by discrete wavelet packet transform (DWPT)**Input:** T1 and T2 Brain ROI images   **Step1:** Apply bior2.2 filter in 2D DWPT to decompose Brain ROI Images   **Step2:** Calculate the selected features according to the transformed coefficients **Output:** Generated nine feature values of each image after 2D DWPT

**Step 8:** Classify brain images.

In the final step, the supervised classifier rough sets will be applied to conduct the classification based on the DWPT feature values and the DCT feature values of the two brain datasets obtained from the TV-seg based algorithm and the proposed hybrid semi-automatic segmentation algorithm. The LEM2 algorithm of the rough sets will be chosen in this study, and the two datasets were classified into two classes: normal and abnormal. Furthermore, the accuracy rate is used to evaluate the classification performance. The calculation of accuracy rate is shown as Equation (11).

(11) $$Accuracy\;=\;\frac{TP+TN}{TP+TN+FP+FN}$$

where:

True positives (TP): with disease and detected as abnormal

True negatives (TN): without disease and detected as normal

False positives (FP): with disease and detected as normal

False negatives (FN): without disease and detected as abnormal

The proposed method will generate eight classification results based on the rough sets LEM2 algorithm, and the other classifiers will be used to compare and verify the proposed method.

## 3. Experiments and Results

In this study, all of the MR images were obtained from The Whole Brain Atlas (Harvard medical school website [26]). Rich brain anatomy information, tutorials and MR images of various diseases are all available on this website. In this study, two brain MR image spectrums, T1 and T2, were used to conduct the experiment. In the T1-weighted images, four types of degenerative disease datasets include T1-weighted images with mild Alzheimer’s disease, T1-weighted images with Alzheimer’s disease, visual agnosia, T1-weighted images with Cerebral calcinosis, T1-weighted images with Pick’s disease and one normal T1-weighted healthy brain image sets that were used to verify the proposed method.

In the T2-weighted images, seven types of degenerative disease datasets include T2-weighted images with mild Alzheimer’s disease, T2-weighted images with Alzheimer’s disease, visual agnosia, T2-weighted images with Cerebral calcinosis, T2-weighted images with Pick’s disease, T2-weighted images with Motor neuron disease, T2-weighted images with Motor neuron disease, T2-weighted images with Huntington’s disease and one normal T2-weighted healthy brain image sets that were used to verify the proposed method. All the brain images selected in this study are the middle slices of the datasets. Table 1 shows the number of normal and abnormal images used in this study.

Two datasets (T1 and T2) were used to conduct the experiments. Forty-two T1-weight brain MR images and 100 T2-weight brain MR images which include normal and degenerative diseases were selected to implement the proposed method. The brain ROI images were obtained from the TV-seg-based segmentation algorithm and the proposed hybrid semi-automatic segmentation algorithm. Next, the feature extraction and feature values acquired from these ROI images by the DWPT and 2D DCT were used as input to the rough sets. For the verification and the comparison, the proposed method generates the eight classification results based on the rough sets LEM2 algorithm. The other three classifiers C4.5 [27], Naïve Bayes [28], and SVM [29] are also used to conduct the classification of MRI normal/abnormal brain tissues for comparison. All of the classifiers in this study will use ⅔images for training and ⅓images for testing with 100 repeated random sampling.

After the experiment, this study compared the classification results of the proposed hybrid segmentation algorithm with the TV-seg segmentation algorithm as Table 2. To discuss the effect of decomposition, the classification results obtained from the proposed 2D DWPT will be compared with the 2D DCT under the two semi-automatic segmentation algorithms as Table 3. In the third column of Table 2 and Table 3, the classification results of the rough sets LEM2 algorithm, the other columns show the results of other classifiers, including C4.5, Naïve Bayes and SVM, which were used to compare with rough sets on the two datasets.

In Table 2, the classification performance of the rough set shows the better classification accuracy and smaller standard deviation compared to the other classifiers. Among all of the combinations of the algorithms in Table 2, the proposed method that combines the hybrid segmentation algorithm and the RS classifier can obtain the best classification performance, the classification accuracy rate is 99.89% and 99.36% on T1 and T2 datasets, respectively, relative to the 94.25% and 98.7% accuracy rate on the TV-seg segmentation algorithm. Based on the classification result of Table 2, the proposed hybrid semi-automatic segmentation algorithm is an available method with a good classification performance, especially as it has a smaller standard deviation.

Table 3 shows the results of different segmentation methods and different decomposition methods (DWPT and DCT). From Table 3, the proposed hybrid segmentation method combined with rough set and 2D DWPT has better accuracy than the other combinations. Based on the experimental results, the DWPT decomposition is better than the DCT.

To further understand if a significant difference exists between the different methods in Table 2 and Table 3, this study used the *t*-test to verify statistically significant differences. In Table 4, the classification performance of the proposed and TV-seg methods shows the significant difference under the T1 and T2 datasets, respectively, and the proposed hybrid segmentation method is better than the TV-seg method in accuracy. Furthermore, when the dataset was analyzed using a different segmentation method, and different decomposition methods (DWPT and DCT), the results also had a statistically significant difference in the T1 and T2 datasets as Table 4. Additionally, the classification accuracy of DWPT was found to be better than the DCT.

## 4. Findings

The experimental results demonstrated the effectiveness of the proposed method. Furthermore, the proposed hybrid segmentation algorithm combined with DWPT and the rough set LEM2 can obtain the best performance. Based on experimental results, several findings are summarized as follows:

1. The proposed hybrid segmentation performance

As shown in Table 2, the proposed hybrid segmentation algorithm performs better than the TV-seg segmentation algorithm, and the proposed method has a smaller standard deviation. In the experimental process, the brain ROI obtained from the two algorithms appeared notably different, and, in the TV-seg segmentation algorithm segment, the image was only based on the shape. Although the proposed hybrid segmentation algorithm had a little over-segment in several images, the pixels of the over-segment area gathered in a different region between normal and abnormal images, and this difference may influence the classification. From the *t*-test results in Table 4, we find that the proposed hybrid segmentation method combined with the wavelet packet and the rough set classifier has better accuracy, because the proposed method has a smaller standard deviation.

2. The DWPT advantage

According to Table 3, the DWPT had a better transformation efficiency compared to the DCT in this study. Due to the DCT transformation, the coefficients of the spatial domain to the frequency domain were based on the block size. Therefore, several images that undergo the DCT transformation may suffer from the transformation problem of “blocking artifact”. However, the DWPT decomposes the image because whole images exclude the interference and obtain better time-frequency transformation. The experimental results of Telagarapu et al. also support this finding [30].

3. The effectiveness of the Rough Set Classifier

Based on Table 2 and Table 3, the rough set LEM2 algorithm is the best classification accuracy and smaller standard deviation among all the classifiers. The rough set could analyze inconsistent and incomplete information, and handles inaccurate and ambiguous situations; therefore, it is more effective than the other classifiers.

4. Dataset quality

The whole classification accuracy of T2 datasets is higher than T1 datasets, because the quality of the T2-weighted images is better than the T1-weighted images. The disease region in the T2-weighted image is clearer than the T1-weighted image. The image with the obvious border is beneficial for segmentation; therefore, the dataset selection and the training of radiologists are also notably important.

## 5. Conclusions

A hybrid semi-automatic segmentation algorithm has been proposed in this study. Additionally, this paper proposed a novel image processing method that combined the wavelet packet and the rough set classifier to verify the proposed segmentation algorithms. Based on the results shown in Table 2 and Table 3, the RS classifier is better than the listing methods in accuracy, and the proposed method is an available technique. From findings in Section 4, to obtain good classification accuracy requires both a suitable method and also image quality. Thus, the training of radiologists is also highly important for obtaining better image datasets. In the suitable method, the proposed method can effectively assist medical staff to identify degenerative brain diseases using T1 images.

An important advantage of the proposed semi-automatic segmentation is that the determination of borders is unbiased and consistent when the brain disease has severe deformable images. Additionally, the proposed semi-automated technique can overcome many segmentation challenges through the use of carefully designed image intensity and anatomical constraints, the results shown by its consistently high accuracy. On the other hand, the proposed hybrid method has some limitations: (1) the proposed hybrid semi-automated segmentation method requires user interaction to initialize region of interest and needs manual time; (2) the proposed method is computationally expensive because the proposed hybrid segmentation method has been combined with the wavelet packet and the rough set classifier.

In the future, the proposed method may be combined with other algorithms to identify other brain diseases, as well as applying the proposed method to other medical image analyses. Additionally, the concept of the proposed method can also be extended to other fields.

## Figures and Tables

**Table 1:** The number of T1-weighted and T2-weighted images.

	T1	T2
Normal	14	40
Abnormal	28	60
Total	42	100

**Table 2:** The results of different classifiers and segmentation algorithms for DWPT.

Dataset	Segmentation Algorithm	RS	C4.5	Naïve Bayes	SVM
T1	Proposed	99.89 (0.33)	89.96 (16.26)	94.99 (6.82)	93.60 (10.20)
T1	TV-seg	94.25 (7.29)	73.01 (9.01)	77.83 (6.41)	66.51 (3.19)
T2	Proposed	99.36 (1.22)	93.53 (3.37)	95.30 (2.85)	95.90 (2.83)
T2	TV-seg	98.7 (2.57)	91.81 (3.28)	91.79 (3.55)	90.94 (4.77)

Note: each numeric cell denotes the average accuracy and the standard deviation in bracket. DWPT, Discrete Wavelet Packet Transform.

**Table 3:** The comparison results for different segmentation and decomposition methods.

Dataset	Method		RS	C4.5	Naïve Bayes	SVM
T1	Proposed	DWPT	99.89 (0.33.)	89.96 (16.26)	94.99 (6.82)	93.60 (10.20)
T1	Proposed	DCT	97.03 (3.8)	85.20 (8.73)	86.54 (7.31)	85.06 (11.04)
T1	TV-seg	DWPT	94.25 (7.29)	73.01 (9.01)	77.83 (6.41)	66.51 (3.19)
T1	TV-seg	DCT	93.65 (6.23)	71.26 (10.79)	78.64 (7.47)	65.75 (3.44)
T2	Proposed	DWPT	99.36 (1.22)	93.53 (3.37)	95.30 (2.85)	95.90 (2.83)
T2	Proposed	DCT	97.79 (2.42)	92.95 (3.01)	95.89 (3.72)	95.02 (3.92)
T2	TV-seg	DWPT	98.7 (2.57)	91.81 (3.28)	91.79 (3.55)	90.94 (4.77)
T2	TV-seg	DCT	97.15 (4.05)	87.69 (5.65)	90.05 (5.65)	86.22 (3.37)

Note: each numeric cell denotes the average accuracy and the standard deviation in bracket.

**Table 4:** The results of pairwise sample *t*-test for difference analysis.

	Proposed vs. TV-seg	DWPT vs. DCT
T1	5.635 ***	7.836 ***
T2	4.488 ***	6.149 ***

Note: *** denotes *p* < 0.01.
